# Introduction to the Special Issue “Biological Efficacy of Natural and Chemically Modified Products against Oral Inflammatory Lesions”

**DOI:** 10.3390/medicines6020052

**Published:** 2019-04-28

**Authors:** Hiroshi Sakagami

**Affiliations:** Meikai University Research Institute of Odontology (M-RIO), 1-1 Keyakidai, Sakado, Saitama 350-0283, Japan; sakagami@dent.meikai.ac.jp; Tel.: +81-49-279-2758 (office); +81-49-279-2787 (M-RIO) (dial-in)

**Keywords:** Kampo medicine, constituent plant extract, stomatitis, oral inflammation, quantitative structure-activity relationship (QSAR) analysis, metabolomics

## Abstract

This editorial is a brief introduction to the Special Issue of “Biological Efficacy of Natural and Chemically Modified Products against Oral Inflammatory Lesions”. From the natural resources and chemical modifications of the backbone structures of natural products, various attractive substances with new biological functions were excavated. Best fit combination of these materials may contribute in the treatment of oral diseases.

Stomatitis is a sore and often recurrent inflammatory condition of the oral mucosa. Approximately 28,542 published papers of stomatitis were found by the recent PubMed search (Survey 1, Table 1). When searched by keyword A (stomatitis) and keyword B (patient’s age), 7695, 10,241, 1142, 2990 and 920 publications were found in elders, adults, young, child and pediatrics, respectively. This indicates that the citing number of older populations in the papers of stomatitis (6695 + 10,241/2 = 8968) were approximately 5.3 times higher than that of younger populations (1142 + 2990 + 929/3 = 1684). Similar trends were found in the papers of herpes stomatitis, aphthous stomatitis, oral inflammation, periodontitis and gingivitis. This may reflect the age-related increase in the incidence of stomatitis.

Oral inflammation is triggered or aggravated by various factors, but how much is each factor involved in the generation of stomatitis? To address this question, the numbers of publications of each factor was searched (Survey 2, Table 1). The most frequently cited factor was virus infection, followed by bacterial infection and radiotherapy. Surprisingly, fungal infection, autoimmune diseases, immune suppression, stress, nutritional deficiency, genetic background, reactive oxygen species (ROS), cigarette and dental prosthetics were cited much less frequently.

The most popular therapeutic agents for stomatitis was searched next (Survey 3, Table 1). Antiviral agents seem to be the most popular for the treatment of stomatitis and herpes stomatitis, followed by antibacterial agents. On the other hand, antibacterial and anti-inflammatory agents appeared to be most often used for the treatment of aphthous stomatitis, oral inflammation, periodontitis and gingivitis. It is surprising that the citation numbers of Traditional Chinese Medicine (TCM) and Kampo medicines were much less, only one tenth that of natural products.

This Special Issue picks up 11 articles, that are listed in four separate sections: Section 1 (Antiviral and antibacterial agents [1,2]); Section 2 (Kampo medicine and constitutive plant extracts [3,4,5,6]); Section 3 (Protection mechanism, [7,8]; (Application of metabolomics and quantitative structure-activity relationship (QSAR) [9,10,11]).

## 1. Antiviral and Antibacterial Agents 

Herpes simplex virus (HSV) infects mainly around the mouth and lips. Epstein–Barr virus (EBV) can manifest in the oral cavity and/or head and neck region. Human papilloma virus (HPV) is often found in oral lesions, including oral hairy leukoplakia (OHL) and/or cancers. Common or notable human immunodeficiency virus (HIV)-related oral conditions include xerostomia (dry mouth), candidiasis, OHL, periodontal diseases, Kaposi sarcoma (KS), HPV-associated warts and ulcerative conditions. Asai and Nakashima stated that among natural sources, the red algal protein GRFT and the algae-derived polysaccharide carrageenan (CG) showed excellent antiviral effects on HIV, HSV-2 and HPV, whereas lignin-carbohydrate (LCC) and sulfated polysaccharide showed the highest anti-HIV activity among natural polyphenols and polysaccharides, by non-specific inhibition of virus adsorption [1].

The human oral cavity is assumed to be a reservoir for the pathogens of many systemic infective diseases. Kanamoto et al. investigated seven *Abiotrophia defectiva*, 17 *Granulicatella adiacens* and six *Granulicatella elegans*, isolated from human oral microbiota for their susceptibility to 15 antimicrobial agents. They found that these bacteria were most sensitive to imipenem and amoxicillin, and there was species-related differences with respect to susceptibilities to ciprofloxacin and minocycline [2].

## 2. Kampo Medicine and Constitutive Plant Extracts

Kampo consists of natural herbs—roots and barks—and has more than 3000 years of history. Watanabe et al. manufactured a Kampo gargle and mastic gel dentifrice for the treatment of peri-implant and severe periodontitis. They found that Kampo reduced the oral bacteria number in vitro, inhibited the bacteria-induced alveolar bone loss and the osteoclast differentiation in vivo, and improved the inflammatory response in the periodontal tissues of patients [3].

Sunagawa et al. reviewed the clinical effect of Hangeshashinto (HST) on cancer patients with chemotherapy-induced mucositis. HST significantly decreased the mean Common Terminology Criteria for Adverse Events grade in the patients and inhibited the growth of Gram-negative bacteria, and the production of PGE_2_ and the expression of COX-2 protein. Its constitutive plant extracts (*Glycyrrhizae* Radix, *Pinelliae* Tuber, *Coptidis* Rhizoma and *Ginseng* Radix) enhanced immunity and increased the activity of natural killer cells in mice [4]. 

Hara et al. showed that among 18 Kampo medicines, HST is most frequently used in Japan, possibly due to the presence of *glycyrrhiza* that contains anti-inflammatory glycyrrhizin. It was surprising that HST had not been used in China [5]. Traditional medicines have been prescribed to elders and adults rather than children, but inclusion of sweet licorice as an ingredient will make it easier for children to take [5].

Ara et al. reviewed the anti-inflammatory action of various natural products [6]. HST inhibited arachidonic acid cascade at multiple points (both COX-1 and COX-2 activities; cPLA2 and COX-2 expressions; ERK phosphorylation). Gingerols and shogaols, the major ingredients in ginger, suppressed NF-κB activation directly or indirectly, leading to the inhibition of COX-2 expression. Recently, β-cryptoxanthin, naringenin, ellagic acid and (-)-epigallocatechin-3-gallate have been reported to show anti-osteoclast characteristics. Since rheumatoid arthritis (RA) is a disease associated with inflammation and bone destruction, and RA prevalence is increased in patients with periodontitis, these natural products may be applicable to treat the periodontitis. 

## 3. Protection and Regeneration Mechanism

Quercetin is a dietary flavonoid found in red wine, tea, many fruits and onions, and well known for its radical scavenging, anti-diabetic, antiviral, anti-pollinosis and anti-allergic activity in vitro and animal models. Edo et al. demonstrated that oral administration of quercetin significantly elevated thioredoxin (TRX) levels in nasal lavage fluids and reduced nasal sneezes and nasal rubbing behaviors. Quercetin’s ability to increase TRX production may account, at least in part, for its clinical efficacy toward allergic rhinitis [7].

Moritani et al. provided a new standpoint of action mechanism of fluocinolone acetonide (synthetic glucocorticoid having 6α,9α-difluoro- and 16,17-acetonide structure) and harmine (one of the β-carboline alkaloids). These compounds strongly induced Cellular Communication Network Factor 2 (CNN2), which encodes protein that is critical in wound healing. Since CNN2 potentiated TGF-β-associated chondrogenesis of bone marrow mesenchymal stem/progenitor cells, harmine may effective to treat stomatitis [8].

## 4. Application of Metabolomics and QSAR

In dentistry, zinc oxide-eugenol formulations have been used for many years as the preferred material for root canal fillings. However, zinc oxide-eugenol released cytotoxic concentrations of eugenol, and induced chronic inflammation. Sakagami et al. demonstrated by metabolomics analysis that Eugenol rapidly induced the vacuolization and suppressed the TCA cycle in human gingival fibroblast, periodontal ligament fibroblast and pulp cells. Similarly, sodium fluoride, that is included in several dentifrices, and benzaldehyde, anticancer principle of figs, blocked the TCA cycle of human oral squamous cell carcinoma cell lines. It is crucial to pursue the biological significance of the inhibition of the TCA cycle in each case for the safe and effective clinical application of these substances [9].

Sakagami et al. reported that among three major natural polyphenols, lignin-carbohydrate complexes (LCC) showed the highest anti-HIV activity, while chemically-modified chromone derivatives (backbone structure of flavonoids) showed much higher anti-tumor activity than most of tannins and flavonoids. QSAR (quantitative structure-activity relationship) analysis suggests a possible link between their tumor-specificity and three-dimensional molecular shape [10]. Although the anti-periodontitis activity of synthetic angiotensin II blockers has been suggested in many papers, natural angiotensin II blockers have not yet been tested for their possible anti-periodontitis activity. Basic structures of the dental plaque is produced from sucrose by glucosyltransferase enzymes (GTFs). Various tannins were found to be excellent inhibitors of GTFs, leading to the manufacture of the gel-entrapped catechin. QASR analysis can be used to explore more selective GTFs and angiotensin II receptor blockers (ARB).

Nagai et al. analyzed the merged data of cytotoxic activities and chemical structures of a total of 494 compounds, and found that the structure-toxicity relationship prediction model showed higher prediction accuracy than the tumor selectivity prediction model. This was mainly due to the fact that descriptors with a high contribution differed for tumor and normal cells. Construction of the tumor selective toxicity prediction model with a higher predictive accuracy may contribute to the screening of candidate compounds for new anticancer drugs [11].

**Figure 1:**
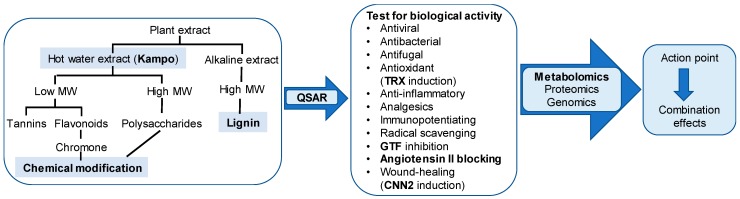
Flow chart to select the best combination of Kampo ingredients.

Natural resources provide numerous useful compounds for treating stomatitis. We can modify their backbone structure to synthesize more active compounds, using QSAR analysis. We can change the combination of candidate components, measure their determined biological activity, and repeat this process until the best combination is determined. Accumulation of such data may lead us to manufacture the best Kampo medicine (Figure 1).

## Figures and Tables

**Table 1:** Survey of publications of stomatitis and related oral inflammations, narrowed down by age-difference, etiology and treatment (based on PubMed search, 13 April 2019).

Keyword B	Number of Cited References					
	Keyword A					
	Stomatitis	Herpes Stomatitis	Aphthous Stomatitis	Oral Inflammation	Periodontitis	Gingivitis
Total	28,542 (100)	1935 (100)	3781 (100)	35,354 (100)	37,596 (100)	13,902(100)
**Survey 1: Age-related citation rate**						
**Elderly**	7695 (27)	349 (18)	1081 (29)	8677 (25)	12,603 (34)	3833 (28)
**Adult**	10,241 (36)	599 (31)	1712 (45)	12,122 (34)	17,833 (47)	6092 (44)
**Young**	1142 (4)	88 (5)	243 (6)	2413 (7)	2603 (7)	1026 (7)
**Child**	2990 (10)	434 (22)	655 (17)	2708 (8)	2339 (6)	2239 (16)
**Pediatric**	920 (3)	83 (4)	235 (6)	1449 (4)	660 (2)	403 (3)
**Survey 2: Etiology**						
**Virus infection**	6267 (22)	1582 (82)	670 (18)	1437 (4)	689 (2)	650 (5)
**Bacterial infection**	1658 (6)	174 (9)	267 (7)	4529 (13)	3852 (10)	1711 (12)
**Radiotherapy**	1521 (5)	46 (2)	54 (1)	352 (1.0)	210 (0.6)	57 (0.4)
**Inflammation**	1181 (4)	53 (3)	164 (4)	N.D.	5989 (16)	2669 (19)
**Autoimmune diseases**	941 (3)	82 (4)	248 (7)	1885 (5)	709 (2)	370 (3)
**Drug-induced**	546 (1.9)	8 (0.4)	22 (0.6)	265 (0.7)	53 (0.1)	485 (3)
**Fungal infection**	1048 (4)	186 (10)	164 (4)	735 (2)	198 (0.5)	214 (2)
**Immune suppression**	428 (1.5)	55 (3)	40 (1.1)	603 (2)	131 (0.4)	98 (0.7)
**Stress**	267 (0.9)	13 (0.7)	92 (2)	2361 (7)	997 (3)	227 (1.6)
**Nutritional deficiencies**	250 (0.9)	9 (0.5)	90 (2)	1243 (4)	110 (0.3)	162 (1.2)
**Genetic background**	202 (0.7)	4 (0.2)	40 (1.1)	240 (0.7)	450 (1.2)	47 (0.3)
**Reactive oxygen species**	119 (0.4)	0 (0)	19 (0.5)	902 (3)	24 (0.06)	150 (1.1)
**Vitamin B deficiency**	107 (0.4)	0 (0)	52 (1.4)	26 (0.1)	2 (0.01)	14 (0.1)
**Cigarette**	60 (0.2)	9 (0.5)	18 (0.5)	250 (0.7)	323 (0.9)	82 (0.6)
**Dental prosthetics**	18 (0.06)	0 (0)	1 (0.03)	19 (0.05)	42 (0.1)	14 (0.1)
**Survey 3: Treatment**						
**Antiviral agent**	3716 (13)	579 (30)	116 (3)	1028 (3)	321 (0.9)	102 (0.7)
**Antibacterial agent**	2333 (8)	86 (4)	324 (9)	3036 (9)	2907 (8)	660 (5)
**Anti-inflammatory agent**	1492 (5)	66 (3)	357 (9)	4975 (14)	1036 (3)	474 (3)
**Steroid**	1234 (4)	59 (3)	319 (8)	2761 (8)	583 (2)	342 (2)
**Natural product**	1105 (4)	85 (4)	120 (3)	2063 (6)	640 (2)	314 (2)
**Trad. Chinese Med.**	54 (0.2)	2 (0.1)	24 (0.6)	349 (1.0)	45 (0.1)	3 (0.02)
**Kampo medicine**	14 (0.05)	0 (0)	1 (0.03)	27 (0.1)	4 (0.01)	2 (0.01)
**Anti-fungal agent**	1029 (4)	48 (2)	55 (1.5)	674 (2)	128 (0.3)	37 (0.3)
**Immunopotentiator**	359 (1.2)	40 (2)	127 (3)	723 (2)	128 (0.3)	37 (0.3)

The top 2 keywords B with high citation rate for each item of Keyword A is highlighted in blue.
